# What to consider when implementing a tool for timely recognition of palliative care needs in heart failure: a context-based qualitative study

**DOI:** 10.1186/s12904-021-00896-y

**Published:** 2022-01-04

**Authors:** Stephanie M. C. Ament, Lisette M. van den Broek, Marieke H. J. van den Beuken-van Everdingen, Josiane J. J. Boyne, José M. C. Maessen, Sebastiaan C. A. M. Bekkers, Louise Bellersen, Hans-Peter Brunner-La Rocca, Yvonne Engels, Daisy J. A. Janssen

**Affiliations:** 1 Department of Health Services Research, Care and Public Health Research Institute (CAPHRI), Faculty of Health, Medicine and Life Sciences, Maastricht University, Maastricht, the Netherlands; 2 Department of Cardiology, Bernhoven Hospital, Uden, the Netherlands; 3 Centre of Expertise for Palliative Care, Maastricht University Medical Centre (MUMC+), Maastricht, the Netherlands; 4 Department of Patient and Care, Maastricht University Medical Centre (MUMC+), Maastricht, the Netherlands; 5 Department of Cardiology, Maastricht University Medical Centre (MUMC+), Maastricht, the Netherlands; 6 Department of Cardiology, Radboud University Medical Centre, Nijmegen, the Netherlands; 7 Department of Anesthesiology, Pain and Palliative Medicine, Radboud University Medical Centre, Nijmegen, the Netherlands; 8 Department of Research and Development, Ciro Horn, P.O. Box 4009, Haelen, 6080 AA the Netherlands

**Keywords:** End-of-life, Palliative medicine, Congestive heart failure, Implementation

## Abstract

**Background:**

Needs assessment tools can facilitate healthcare professionals in timely recognition of palliative care needs. Despite the increased attention for implementation of such tools, most studies provide little or no attention to the context of implementation. The aim of this study was to explore factors that contribute positively and negatively to timely screening of palliative care needs in advanced chronic heart failure.

**Methods:**

Qualitative study using individual interviews and focus groups with healthcare professionals. The data were analysed using a deductive approach. The Consolidated Framework for Implementation Research was used to conceptualise the contextual factors.

**Results:**

Twenty nine healthcare professionals with different backgrounds and working in heart failure care in the Southern and Eastern parts of the Netherlands participated. Several factors were perceived to play a role, such as perception and knowledge about palliative care, awareness of palliative care needs in advanced chronic heart failure, perceived difficulty when and how to start palliative care, limited acceptance to treatment boundaries in cardiology, limited communication and collaboration between healthcare professionals, and need for education and increased attention for palliative care in advanced chronic heart failure guidelines.

**Conclusions:**

This study clarified critical factors targeting patients, healthcare professionals, organisations to implement a needs assessment tool for timely recognition of palliative care needs in the context of advanced chronic heart failure. A multifaceted implementation strategy is needed which has attention for education, patient empowerment, interdisciplinary collaboration, identification of local champions, chronic heart failure specific guidelines and culture.

**Supplementary Information:**

The online version contains supplementary material available at 10.1186/s12904-021-00896-y.

## Introduction

Timely providing palliative care to patients with advanced chronic heart failure (CHF) improves patient outcomes, patient satisfaction, documentation of care preferences and cost utilization [1, 2]. Following the recent statements of the Heart Failure Association of the European Society of Cardiology and the European Association for Palliative Care, palliative care must be accessible for all patients with CHF, regardless of their prognosis [3, 4]. Although the recognition of the relevance of palliative care is growing, palliative care is not yet routinely integrated in current advanced CHF care [5]. If provided, patients with advanced CHF often receive palliative care very late in the course of advanced CHF [6, 7].

Due to the unpredictable course of the disease, appropriate recognition of palliative care needs in advanced CHF is difficult [8, 9]. To facilitate timely recognition of patients with CHF in need for palliative care, an approach focusing on identification of palliative care needs is more appropriate than the recognition of a poor prognosis [3, 10]. Tools such as the ‘Needs Assessment Tool: Progressive. Disease – Heart Failure’ (NAT-PD:HF) [11, 12], the ‘Integrated Palliative Care Outcome Scale’ (IPOS) [13] and the ‘Identification of patients with HeARt failure with Palliative care needs’ (I-HARP) [14] have been developed to facilitate healthcare professionals in timely recognition of palliative care needs in advanced CHF The recently developed Question Prompt List is an example of a needs-specific tool which specifically facilitates the recognition of information needs about advance care planning [15].

Theoretically, application of palliative care needs assessment tools in the care process of patients classified with New York Heart Association (NYHA) class III or IV may lead to more value-driven and person-centred care [16, 17]. However, implementation of new routines and practices in healthcare is a complex process [18, 19]. Consequently, successful implementation does not happen spontaneously, even in the presence of solid evidence. Understanding the context using a whole system approach focusing on the individuals, organisations and external policies and trends should already be addressed in the early implementation stage, when stakeholders are aware that change is needed [20]. Different factors in the pre-implementation, in the early and late implementation and in the post-implementation phase influence the level of successful change [21]. To increase the chance for successful implementation, insight into these factors is essential to adequately design a tailored implementation strategy to overcome context-based barriers [22, 23]. Proctor et al. defined an implementation strategy as “methods or techniques used to enhance the adoption, implementation and sustainability of a clinical program or practice” [24]. To our knowledge, few research has been performed into the implementation of needs assessment tools to screen for palliative care needs in advanced CHF [17, 25].

Therefore, the aim of the current study was to explore the factors that contribute positively and negatively to the integration of timely recognition of palliative care needs in the context of advanced CHF as perceived by healthcare professionals (HCPs) in the Dutch CHF care. These insights are relevant for the implementation activities that may be needed in combination with a palliative care needs assessment tool.

## Methods

### Design

A descriptive qualitative research design was chosen to comprehensively describe the research phenomenon [26]. The current study is part of the project “Identification of patients with HeARt failure with Palliative care needs (I-HARP)” (2018–2021). The aims of the I-HARP project are to develop and implement a needs assessment tool to timely recognize palliative care needs in advanced CHF [14]. The Consolidated Criteria for Reporting Qualitative Research guidelines were used to ensure transparency and improve rigour [27].

### Needs assessment tools: I-HARP and NAT-PD:HF

I-HARP is a general and accessible tool for HCPs and is promising to facilitate timely recognition and directing of personal palliative care needs in CHF during a conversation. The development of I-HARP has been described elsewhere [14]. Before the development of I-HARP, heart failure nurses piloted a Dutch translation of the NAT:PD-HF during a usual care home visit with the patient with advanced CHF [28]. The pilot study revealed that the NAT:PD-HF was not acceptable and usable for the heart failure nurses in the Dutch setting. The NAT:PD-HF showed to lack questions helping to communicate about palliative care, the questions were perceived to not fit with the patient’s needs and the tool was perceived to be too complex in use.

### Participants

HCPs caring for patients with advanced CHF were asked to particpate. They were recruited using criterion and snowball sampling which was based on age, gender, type of healthcare organization, discipline, treatment setting (inpatient/ outpatient) and the level of expertise in palliative care [29]. HCPs were recruited in six general practices and two hospitals in the Southern and Eastern parts of the Netherlands. The sampling plan was developed by SA, DJ, JB and MvB.

### Data collection

Focus groups, interviews, and fieldnotes were used for data collection. One focus group was held with heart failure nurses who were involved in a pilot study using the NAT:PD-HF in the home setting in 2016 [28]. The aim of this focus group was to explore barriers and facilitators towards palliative care needs assessment in general and to evaluate the possible integration of the NAT:PD-HF during a home-based consultation. All other data collection took place between November 2018 and March 2019 before the development and implementation of I-HARP [14]. All respondents in both studies were aware of the aim to develop, refine and implement a needs assessment tool to facilitate healthcare professionals in timely recognition of palliative care needs. The topic list for the focus groups and interviews were developed by the research team with backgrounds in cardiology (JB, IC), palliative care (MvB, DJ), old age medicine (DJ) and implementation science (SA) (supplementary file 1). The interviews were performed by a member of the research team (SA). A moderator and an observer were involved in each focus group (SA, DJ, JB, IC). Each interview and focus group started with an introduction, including the aim of the meeting, the research project and the rules of the interview. Interviews and focus groups were recorded and transcribed verbatim.

### Data-analysis

The Consolidated Framework for Implementation Research (CFIR) [21, 23] was used as a systematic approach gathering and analyzing data deductively regarding the factors related to integration of timely recognition of palliative care needs as perceived by HCPs [30]. Before the development of I-HARP, we explored the desired characteristics of a future needs assessment tool using the ‘intervention characteristics’ domain of the CFIR. These data were published before [31]. In this study, we focused on the context of integrating timely recognition of palliative care needs and focused on the characteristics of the inner setting (inter-organizational networks, communication, implementation climate and organizational culture), the outer setting (patient needs, external policies, trends and financial incentives) and of the individuals involved (knowledge and beliefs, learning stage, personal attributes and self-efficacy of HCPs). The framework was used to direct the content of the interview guide and to guide the coding process of the interviews. Data-analyses started directly after the first interview using direct content analysis [32]. Qualitative data were coded independently by SA (implementation researcher, involved in the coding of all data), WE (general practice-based nurse, coding of the primary care collected data) and IC (heart failure nurse, coding of the hospital collected data). Agreement on the coding was reached during consensus meetings. The interview with the palliative care consultant and the focus group with heart failure nurses who piloted the NAT-PD:HF were used to check for datasaturation.

### Ethical considerations

The medical ethical committee of the Maastricht University Medical Centre (MUMC+), Maastricht, the Netherlands approved the study protocol and concluded that the study was not subject to the Medical Research Involving Human Subjects act (2018–0638). All participants provided written informed consent.

### Trustworthiness

Trustworthiness was pursued by applying strategies regarding credibility and transferability [33]. Credibility was established by data triangulation, i.e. working with interview data, focus group data and fieldnotes, investigator triangulation and analyst triangulation, i.e. working with different researchers as moderators and observers during focus groups and analysing sections of the data with researchers with different backgrounds. Also, the interpretations of the data were checked by sending the participants a summary after each session (member check).

We used thick description as a way to enable the reader to make a transferability judgment. Thick description is a technique in which a qualitative researcher provides a robust and detailed account of their experiences during data collection [33]. Next to this, extensive field notes of the interviews and focus groups were written.

## Results

Three HCP focus groups (*n* = 8, *n* = 6, n = 8) were held in two different hospitals. Mean duration of the focus groups was 104 (range 99–110) minutes. Four family physicians, two general practice-based nurses and one palliative care consultant working at the hospital were interviewed by phone as they were unable to attend a focus group. Mean duration of the individual interviews was 47 (range 31–54) minutes. Baseline characteristics of the HCPs are presented in Table 1. Factors related to timely identification of palliative care needs in advanced CHF were found in constructs of the CFIR.

**Table 1:** Characteristics of participating healthcare professionals (*n* = 29)

Male gender (n, %)	5 (17%)
Age (mean, range)	48 (25–66)
*Profession (n, %)*	
Cardiologist	4 (14%)
Heart failure nurse specialist	11 (38%)
General practitioner	4 (14%)
General practice-based nurse	2 (7%)
Palliative care specialist/consultant	2 (7%)
Registered nurse	3 (10%)
Certified nurse assistant	1 (3%)
Team leader	2 (7%)
Specialized in palliative care^a^	4 (14%)

^a^Finished a general practitioner or medical specialization in palliative care, obtained a PhD degree in palliative care or finished higher education palliative care

### Characteristics of the inner setting

Perceived factors related to the inner setting of the organization were ‘networks and communication’, ‘compatibility’ and ‘culture’ [21].

#### Networks and communication

‘Networks and communication’ refers to the nature and quality of webs of social networks and the nature and quality of formal and informal communications within an organization [21].

Limited communication about patient’s care within a team and within the (inter)disciplinary network was perceived as a barrier for timely recognition of palliative care needs. More information about what was discussed with the patient after a consultation, interdisciplinary meetings and structural planning of consultations were mentioned to influence timely recognition of palliative care needs.

*‘Once you’ve raised things with a patient and you notice that you’re not getting anywhere, that it’s difficult or that it’s not going easily, that in particular deserves multidisciplinary consultation. With the GP, the palliative team and the spiritual counsellor, because it can be very difficult at times.’ (Heart failure nurse)*

Clear roles and collaboration with respect to recognizing and discussing palliative care needs were perceived as enablers. The general practice-based nurses and heart failure nurses were mentioned to have a more signalizing role related to palliative care needs since they have relatively more contact moments and time with the patient than the physicians, such as cardiologists.

*‘I think you [heart failure nurses] are the ones who notice much more easily than we do. I don’t pick up on it. That’s the unfortunate consequence of having only 10 minutes per patient. But when you notice and you sense that it’s not going well, that’s the moment to decide together with the treating cardiologist that we need to do something about this.’ (Cardiologist)*

A relation of trust between the HCP and the patient was mentioned as an enabler for better understanding of what the patient actually needs.

*‘What’s also important to us is to have those patients come back regularly to someone they know and not to different people, because that happens regularly as well for scheduling reasons. Trust is important in the relationship too.’ (Heart failure nurse)*

#### Compatibility

‘Compatibility’ refers to the degree of tangible fit between meaning and values attached to the intervention by involved individuals, how those align with individuals’ own norms, values, and perceived risks and needs, and how the intervention fits with existing workflows and systems [21].

Respondents working at the hospital mentioned that they have to push to claim moments or consultations to recognize palliative care needs. Respondents mentioned that having limited time sometimes was an excuse for not talking about palliative care needs.

*‘Today’s workplace culture in hospitals simply doesn’t allow discussing these kinds of things – especially since doctors are not very comfortable talking about it anyway. Having too little time then becomes a very easy excuse not to do it.’ (Cardiologist)*

#### Culture

‘Culture’ refers to the norms, values and basic assumptions of a given organization [21].

On discipline level, cardiology was perceived as a discipline with a culture which is focused on treatment and adherence to clinical practice guidelines with respect to the disease CHF to optimize survival, which was also seen as a barrier.

*‘When I came back from internal medicine rotation, where I’d spent most of my time on the geriatric ward, I really noticed the difference. I’ve found that we are very much focused on curative care, whereas they thought more in terms of holistic domains.’ (Cardiologist)*

Respondents mentioned working in the hospital mentioned that they do not feel comfortable to make time for conversations with patients about palliative care needs.

### Characteristics of the outer setting

Perceived factors related to the outer setting of the organization revealed to be ‘patient needs and resources’ and ‘external policies and incentives’.

#### Patient needs and resources

‘Patient needs and resources’ refers to the extent to which patient needs, as well as barriers and facilitators to meet those needs, are accurately known and prioritized by the organization [21].

General practitioners and general practice-based nurses perceived that due to innovations in CHF care, their CHF patient case-mix becomes more complex. HCPs working in primary care mentioned a patients delay with respect to timely recognition of palliative care needs due to the fact that patients with CHF adapt to their limitations and don’t call for help on time.

‘*These people don’t speak up much either. I always notice that. You can also see that when you analyze the demand for care of someone going through heart failure, which often is only focused on when acute care is needed.’ (General practitioner)*

Respondents mentioned that patients with advanced CHF often receive care from the cardiology department and to a lower extent from the general practitioner. The consultant palliative care and a general practitioner mentioned that the majority of patients with CHF has faith and trust in the cardiologist’s opinion.

*‘When they go to see their GP, people often cling very strongly to the opinion of the cardiologist, who is the specialist and has to support things.’ (Consultant palliative care)*

All respondents mentioned that patient information about advanced CHF and palliative care is an enabler for timely recognition of palliative care needs.

*‘You have to help the patient to get the right information. Otherwise, you don’t know if the information they’re getting is giving them realistic expectations, because that’s where it starts. You have to know what they understand and what they can expect.’ (Cardiologist)*

#### External policies and incentives

‘External policies and incentives’ refers to a broad construct that includes external strategies to spread interventions, including policy and regulations (governmental or other central entity), external mandates, recommendations and guidelines, pay-for-performance, collaboratives, and public or benchmark reporting [21].

Respondents working in the hospital mentioned that future developments in e-health could facilitate timely recognition of palliative care needs.

*‘I think telephone or video call consultations could help a lot too. If you use it to prepare for an outpatient consultation, you can already bring up certain difficult points and to see if there’s a need to discuss a certain topic. Then you also have your opening to address something.’ (Cardiologist)*

A general practitioner mentioned that the integration of palliative care in general takes time. Cardiology was mentioned as a discipline that falls behind compared to for example oncology and pulmonology.

*‘I think in oncology, where they’ve been doing palliative care for a lot longer, they’re much more aware that the symptom burden in COPD is similar, which only just now is starting to be reflected in the guidelines. Such awareness comes much later when it’s heart failure or COPD …’ (General practitioner)*

### Characteristics of the individuals

Perceived factors related to the individuals were ‘knowledge and beliefs’, ‘individual stage of change’, ‘personal attributes’ and ‘self-efficacy’ [21].

#### Knowledge and beliefs

‘Knowledge and beliefs’ refers to individuals’ attitudes toward and value placed on the intervention as well as familiarity with facts, truths, and principles related to the intervention [21].

In all focus groups, respondents discussed their knowledge and beliefs about palliative care, to be sure that they all discussed the same issues. Many respondents perceived difficulties in describing the concept of palliative care and perceived the tendency to associate it with difficult conversations and terminal care.

*‘When we talk about palliative care, do we all mean the same thing?’ (Cardiologist 1)*

*‘I think it involves much more than what we mean by it.’ (Cardiologist 2)*

#### Individual stage of change

‘Individual stage of change’ refers to the characterization of the phase an individual is in, as he or she progresses toward skilled, enthusiastic, and sustained use of the intervention [21].

Some respondents mentioned examples of tools that can help in assessing palliative care needs. A consultant palliative care perceived that years of work experience without a palliative mindset strongly limits the HCP’s stage of change. Some respondents mentioned the importance of integration of a palliative mindset into general education. Two general practitioners mentioned that they recognize an early change in their field with respect to awareness and knowledge about palliative care needs in non-oncological patients such as patients with advanced CHF.

‘*I feel that people are slowly starting to understand that this also concerns other patients than just the oncology ones. GPs and cardiologists are starting to realise that patients feel a need for this, and that they are palliative patients too.’ (General practitioner)*

#### Personal attributes

‘Personal attributes’ refers to a broad construct to include other personal traits such as tolerance of ambiguity, intellectual ability, motivation, values, competence, capacity, and learning style [21].

The abilities of general practice-based nurses to recognize palliative care needs were mentioned by a general practitioner to be promising but very variable due to differences in educational background. Some respondents mentioned that heart failure nurses were educated to deliver protocol-based care (for example focus on weight, kidney function and diuretics) which could hinder the recognition of palliative care needs. Respondents working in the hospital mentioned the good communication skills of some spiritual caregivers in relation to recognition of palliative care needs.

*‘I sometimes call the spiritual counsellor and say “I’m sitting here with a patient and I’m not getting anywhere”. And he says: “Schedule a consultation, I’ll be right up.” Most patients say: “He’s so calm and pleasant to talk to. He understands me, and I don’t even know him.” He also has a way with asking questions. It really astounds me. I’d like to have that gift myself.’ (Nurse)*

#### Self-efficacy

‘Self-efficacy’ refers to the individual belief in their own capabilities to execute courses of action [21].

The perceived self-efficacy to recognize palliative care needs was limited due to the grey area of when to start the conversation about palliative care. Some respondents perceived difficulties with having conversations about palliative care.

*‘But it’s a tricky thing to do, asking patients what they want. To me, patients don’t always seem very clear about what they want. Actually, people usually – or at least quite a few of them – want to stick their head in the sand and just want to get better.’ (Heart failure nurse)*

## Discussion

The current study explored factors related to the integration of timely recognition of palliative care needs in the context of advanced CHF as perceived by healthcare professionals (HCPs) in the Dutch CHF care. Characteristics of the inner setting, characteristics of the outer setting and characteristics of the HCP were perceived to play a role in the integration of timely recognition of palliative care needs.

### Inner setting

This study showed that limited time, limited communication and limited collaboration between HCPs were perceived as barriers for timely recognition of palliative care needs in advanced CHF. Collaboration and sharing information is needed to make timely recognition of palliative care needs more efficient and compatible [4]. Research revealed that team work regarding advanced CHF and palliative care is the most promising strategy to improve patient-centered outcomes [1, 34]. Specification of responsibilities and roles on all levels of the interdisciplinary organization and having a professional responsible increase the chance for sustainable integration of timely recognition of palliative care needs [35]. Furthermore, efforts need to be undertaken to invest in a culture in which making time for timely recognition of palliative care needs is valued and respected as care as usual. Recruitment and training of clinical leadership among this topic may help to change the culture in cardiology [36]. Timely recognition of palliative care needs must be adopted in the organization’s palliative care values and work agreements to facilitate sustainable integration, preferably in a learning environment with feedback [35]. HCPs working in advanced CHF care may also learn from close collaboration with palliative care specialists.

### Outer setting

Developments in e-health in the context of heart failure were perceived to be an enabler for timely identification of needs. Providing information and asking explorative questions about palliative care needs using e-health may facilitate patient empowerment and the participatory role of the patients with advanced CHF [37]. Patients may have more time to reflect on issues which supports the patient/ HCP dialogue [38]. However, many patients with CHF and family members prefer recognition of palliative care needs during a conversation [31]. The current study revealed that especially HCPs working in the primary care experience difficulties due to the complexity of this patient group. Patients with advanced CHF have palliative care needs and more proactive consultations in primary care are needed to clarify these needs, whishes, values and desires [39]. HCPs working in primary care are focused on care delivery for the whole person instead of the disease. However, to help the general practioner identifying patients with advanced CHF and adopting a palliative care approach, the cardiologist or heart failure nurse must notify the general practitioner in time. The consultative role of cardiology in primary care may be improved due to the experiences with virtual interdisciplinary communication during the Covid-19 pandemic. Interdisciplinary champions working with a virtual collaborative structure need to be identified, who dedicate themselves to supporting, marketing, and driving through an implementation process, overcoming indifference or resistance in the current context of advanced CHF and palliative care [40]. If established digital structures are proven, financial incentives could be developed to facilitate sustainability and spread of change.

### Individuals

The current study showed that HCPs perceive different barriers to recognize palliative care needs. HCPs working in CHF care need to be aware of the value of palliative care and need more education and experience in palliative care. Organizing educational meetings, educational outreach visits by an expert, training sessions, creating a learning collaborative and developing educational materials are strategies that could be used to improve the knowledge among HCPs [36]. A general and accessible semistructured clinical interview guide for comprehensive needs assessment may facilitate HCPs in practice [14]. Currently, palliative care is integrated in palliative care specific guidelines [3, 41]. More attention to palliative care in heart failure-specific clinical practice guidelines is needed to implement palliative care in current CHF care and to remind HCPs of palliative care. However, standardization is not enough to integrate timely recognition of palliative care needs in CHF care [42]. Making use of discipline-specific talents and strengths for timely recognition, especially on general practice-based nurse and heart failure nurse level, is needed to provide value-driven and person-centred care [43, 44].

### Future directions

Future research would benefit from insight in what patients with advanced CHF need to empower them before consultation. Patient education or reflective questions using e-health may facilitate timely recognition of palliatve care needs in advanced CHF. Moreover, quality improvement initiatives focusing on implementing a tool for timely recognition of palliative care needs in advanced CHF must use these insights before the start of an implementation project. A tool such as I-HARP is merely focused on the behavior of the HCP (individual) and the current study shows a need for a change strategy targeting the HCP, the inner and the outer setting of the healthcare organization. Quality improvement initatives may refine and prioritize the actions needed with patient representatives and local and national stakeholders. Small scale local pilots could be used to test and adapt the implementation strategy components for possible spread to other contexts [45].

### Strengths and weaknesses

An important strength of this study is the use of the evidence-based CFIR framework which is specific and detailed concerning contextual implementation determinants [21, 46]. Few studies used this framework in the pre-implementation phase [23]. Another strength is the use reflective feasibility evaluation of the NAT:PD-HF combined with the explorative data that was collected as part of the development process of I-HARP. Some limitations of this study should be addressed. Patients with advanced CHF and their family caregivers were not included as stakeholder in this research. Nevertheless, their viewpoints regarding the desired tool’s characteristics for timely identification of palliative needs in advanced CHF were published before [31]. Patient engagement can help to create a receptive context for quality improvement [47].

## Conclusions

The integration of timely recognition of palliative care needs in patients with advanced CHF and their family is still in its infancy. A single-faceted implementation strategy is not enough to implement a needs assessment tool in the context of heart failure and palliative care. Possible components to include in an implemenation strategy are: focus on better communication and collaboration between HCPs, patient empowering strategies, e-health, proactive consultations in the primary care setting triggered by the hospital, identification of champions and training leaderschip, education strategies for HCPs and attention for palliative care in heart failure specific guidelines and protocols. Healthcare organizations, HCPs and policymakers must use these insights when preparing for integration of a palliative care needs assessment tool in advanced CHF.

## Supplementary Information

**Additional file 1.** Topic List.

## Data Availability

The datasets generated and analysed during the current study are not publicly available due to the confidentiality and the traceability of the qualitative data but are available from the corresponding author on reasonable request.

## Abbreviations

CHF: Chronic heart failure
HCP: Healthcare professional
