# Supplementary material for: What to consider when implementing a tool for timely recognition of palliative care needs in heart failure: a context-based qualitative study
Source: BMC Palliat Care. 2022 Jan 4;21:1. doi: 10.1186/s12904-021-00896-y (PMC8723899; doi:10.1186/s12904-021-00896-y)
Supplement: Supplementary file 1 — Additional file 1. Topic List. [file 12904_2021_896_MOESM1_ESM.docx]

**Supplementary file 1. Core questions for the interviews and focus groups**

How would you describe the current situation within your organisation with respect to timely recognition of palliative care needs in patients with advanced CHF?

How would you describe your experiences for timely recognition of palliative care needs in patients with advanced CHF? Could you give an example?

What makes it difficult to recognize palliative care needs in patients with advanced CHF?

Imagine that a patient explicitly mentions to have palliative care needs, what would be a barrier to explore and direct this palliative care need.

What would you need for timely recognition of palliative care needs in patients with advanced CHF?

What would you need for directing the palliative care needs in patients with advanced CHF?
